# Efalizumab in the Treatment of Scalp, Palmoplantar and Nail Psoriasis: Results of a 24-Week Latin American Study

**DOI:** 10.1111/j.1753-5174.2009.00025.x

**Published:** 2010-03

**Authors:** María Denise Takahashi, Edgardo Néstor Chouela, Gladys Leon Dorantes, Ana Maria Roselino, Jesùs Santamaria, Miguel Angel Allevato, Tania Cestari, Maria Eugenia Manzanera de Aillaud, Fernando Miguel Stengel, Daiana Licu

**Affiliations:** * Faculdade de Medicina de USP, Hospital das ClínicasSão Paulo, Brazil; † Hospital General de Agudos “Dr Cosme Argerich”Buenos Aires, Argentina; ‡ Hospital General de MexicoMexico City, Mexico; § USP Ribeirão PretoSão Paulo, Brazil; ¶ Universidade Federal do ParanáParaná, Brazil; ** Hospital de Clínicas “José de San Martín”Buenos Aires, Argentina; †† Universidade Federal de Porto Alegre, Hospital de ClínicasPorto Alegre, Brazil; ‡‡ Clínica 20 IMSSTijuana, Mexico; §§ Centro de Educacion Médica e Investigaciones (CEMIC), Clinicas “Norberto Quirno”Buenos Aires, Argentina; ¶¶ Merck Serono International S.A., Geneva, Switzerland, an affiliate of Merck KGaADarmstadt, Germany

**Keywords:** Efalizumab, Nail, Palmoplantar, Psoriasis, Scalp

## Abstract

**Introduction:**

Plaque-type psoriasis affecting the nails, scalp, hands or feet can often be difficult to treat; for example, topical treatments and phototherapy may not penetrate the nail plate or scalp. The objective of this large, international, multicentre study was to investigate the efficacy of efalizumab in a Latin American population of adult patients with moderate-to-severe chronic plaque psoriasis who were candidates for systemic therapy or phototherapy.

**Methods:**

Eligible patients were enrolled in a 24-week, open-label, single-arm, Phase IIIb/IV study of continuous treatment with subcutaneous efalizumab, 1.0 mg/kg/wk. Involvement of the nails, scalp, or hands or feet was assessed using the Nail Psoriasis Severity Index (NAPSI), the Psoriasis Scalp Severity Index (PSSI), or the Palmoplantar Pustulosis Psoriasis Area and Severity Index (PPPASI), respectively. Missing data were handled using a last observation carried forward or nonresponder imputation approach.

**Results:**

Of the 189 patients who received treatment, 112 patients had nail involvement, 172 had scalp involvement, and 19 had palmoplantar disease at baseline. At Week 24, ≥50% improvement on the NAPSI, PSSI and PPPASI was observed in 31%, 71% and 68% of patients, respectively, whereas ≥75% improvement on these scores was observed in 17%, 52% and 63%, respectively. Descriptive statistics showed lower NAPSI-75 and higher PSSI-75 and -50 response rates among patients with higher baseline scores.

**Conclusions:**

This open-label, uncontrolled study provides supportive evidence of the potential of efalizumab as a treatment for nail, scalp and palmoplantar psoriasis.

## Introduction

Psoriasis is a chronic, autoimmune, T-cell-mediated inflammatory skin disorder that affects various parts of the body [1]. Psoriasis occurs in approximately 1–3% of the population in Europe and the USA [2–4]. The nails, scalp, palms and soles of the feet are especially problematic sites in the everyday treatment of psoriasis [5]. In particular, the nails and scalp are not amenable to many topical treatments or phototherapy because of the impermeability of the nail plate and the protective effects of hair on the scalp [6–8].

Efalizumab is a recombinant, humanized, monoclonal, immunoglobulin G1 antibody that binds specifically to the CD11a subunit of leucocyte function-associated antigen-1, inhibiting major steps in the immunopathogenesis of psoriasis: T-cell activation, migration and reactivation [9]. The efficacy and safety of efalizumab 1.0 mg/kg/wk in the treatment of moderate-to-severe plaque psoriasis have been demonstrated in an extensive clinical trial programme conducted in Europe and North America [10–15]. At the time this study was completed, efalizumab was approved for the treatment of adults with moderate-to-severe, chronic plaque psoriasis.

In previous studies of efalizumab, efficacy was assessed using the Psoriasis Area and Severity Index (PASI); no previous detailed analyses of the efficacy of this agent in the treatment of nail, scalp and palmoplantar psoriasis have been published to date. This multicentre, open-label, Phase IIIb/IV trial examined the efficacy of efalizumab in a population of patients with psoriasis in Latin America; primary efficacy and safety results were presented by Stengel et al. at the 2008 5th Spring Symposium of the European Academy of Dermatology and Venereology and are reported elsewhere [16]. Here, we present analyses of data from subgroups of patients with nail, scalp or palmoplantar psoriatic involvement at study entry.

## Methods

This was a 24-week, multicentre, open-label, single-arm, Phase IIIb/IV study (protocol IMP25161; ClinicalTrials.gov registration NCT00287118) conducted between October 2004 and May 2006 in 23 centres in Latin America (five in Argentina, nine in Brazil, and nine in Mexico). The reader is referred to Stengel et al. (this volume) for a complete briefing of the methodology.

### Patients

Patients were between 18 and 75 years of age and had moderate-to-severe plaque psoriasis (≥10% body surface area involved and candidates for systemic therapy or phototherapy). Discontinuation of any systemic psoriasis treatment was required prior to commencement of the trial; in the case of biologics, a 3-month washout period was required. For women of childbearing potential and for men whose partners could become pregnant, consent to use an acceptable method of contraception and agreement to continue to practise an acceptable method of contraception for the duration of their participation in the trial and up to 3 months after the last dose of efalizumab, were mandatory for study participation. The dosage of any medications required for treatment of comorbidities must have been stable for at least 28 days before the administration of study drug. All patients underwent initial screening ≤14 days prior to the first efalizumab injection. Discontinuation of any systemic psoriasis treatment was mandatory before starting study medication; no washout period was required. Patients included in the subgroup analyses had baseline scores > 0 on the Nail Psoriasis Severity Index (NAPSI) [17], indicating nail involvement at baseline; the Psoriasis Scalp Severity Index (PSSI) [18], indicating scalp involvement; or the Palmoplantar Pustulosis Psoriasis Area and Severity Index (PPPASI) [19], indicating involvement of the palms and/or soles. Key exclusion criteria included guttate, erythrodermic or pustular psoriasis as the sole or predominant form of psoriasis. Patients were also ineligible if they had active disease rebound during or following discontinuation of previous efalizumab treatment (i.e. a PASI >125% from baseline and/or new predominant morphology of psoriasis) if this outcome was related to efalizumab adverse events or related to lack of efalizumab efficacy; however, if active disease rebound was related to a nondrug reason (e.g. infection or vaccination) then patients were eligible for study drug medication. The study was carried out in compliance with the Declaration of Helsinki and Good Clinical Practice guidelines, and the protocol was approved by the research ethics committees of the centres involved. Patients were informed of the objectives and overall requirements of the study, and all gave written informed consent.

### Treatment

All patients received open-label efalizumab administered subcutaneously, starting with an initial conditioning dose of 0.7 mg/kg at baseline (study day 0), followed by 23 weekly doses of efalizumab 1.0 mg/kg. Injection sites were rotated on a weekly basis and efalizumab was self-administered by patients after the first injection.

### Efficacy Assessments

The primary efficacy endpoint in the parent trial was the proportion of patients with a rating of “excellent” or “cleared” at Week 24 using the dynamic Physician Global Assessment (PGA), a tool that measures the response of all psoriatic lesions to therapy by comparing the subject's present condition to baseline photographs or body diagrams. The assessor classified response by considering erythema, scaling, plaque thickness and percentage of body surface area affected. Ratings of cleared and excellent represented a 100% improvement (remission) and 75% to 99% improvement of all clinical signs and symptoms relative to baseline, respectively. The nail, scalp and palmoplantar endpoints, which were tertiary endpoints in the parent trial, are presented in detail here. The median percentage improvements over time in NAPSI, PSSI and PPPASI scores are reported for the subgroups of patients who had baseline scores > 0 for these indices, as well as the percentages of patients showing at least a 50% and 75% improvement in these scores between baseline and Week 24.

Assessment of nail psoriasis was made using the NAPSI (score range 0–80, with higher scores indicating greater involvement; all involved nails are assessed), at baseline and at Weeks 12 and 24. Assessment of scalp psoriasis was made using the PSSI (score range 0–72, with higher scores indicating more severe disease). Palmoplantar psoriasis was assessed using the PPPASI (score range 0–72, with higher scores indicating more severe disease). Both the PSSI and PPPASI were measured at baseline and at Weeks 4, 8, 12 and 24 (or at the time of last dose).

### Statistical Analysis

Analyses of efficacy in the intent-to-treat (ITT) population were performed using the last observation carried forward (LOCF) approach. The primary endpoint was also analysed using the nonresponder imputation, in which all patients with missing data at Week 24 were counted as nonresponders. No formal hypothesis testing or adjusted analyses were performed. Analysis was performed using SAS software (SAS Institute, Inc., Cary, NC, USA).

The efficacy data reported here for nail, scalp and palmoplantar psoriasis are for the subsets of patients who had scores on the NAPSI, PSSI and PPPASI that were >0 at baseline (i.e. patients who had some nail, scalp or palmoplantar disease at baseline). Demographic and treatment data are for the entire ITT population. Summary statistics were determined for scores; 95% confidence intervals (CIs) for the median were estimated using the exact method based on the binomial distribution. For proportions, exact CIs were used. The analysis of patients showing 50% and 75% improvement in NAPSI, PSSI and PPPASI scores was *post hoc*.

## Results

### Demographic and Disease Characteristics

Overall, 189 patients were enrolled and received treatment (ITT population), of whom 137 (72.5%) completed the 24-week treatment period (Table 1). A total of 30 and 11 patients discontinued treatment due to adverse events and lack of efficacy, respectively. Eleven other patients did not complete the treatment course for a range of other reasons, including loss to follow-up and protocol violations. These factors, as well as failure to record all patient data sets (and their associated statistical descriptors), accounted for most of the missing datapoints. The median (range) age in the ITT population was 46 (19–74) years and two-thirds of patients enrolled were men. Hispanics or Latinos comprised the majority ethnic group in this population (104/189 [55%]). A total of 112 patients had nail involvement, 172 had scalp involvement and 19 had palmoplantar involvement at baseline; 1 of these 19 patients had pustules at baseline.

**Table 1:** Summary of baseline patient demographic and disease characteristics

Characteristic	ITT population (N = 189)
Age in years, median (range)	46 (19–74)
Male sex, n (%)	134 (70.9)
Race, n (%)	
White	125 (66.1)
Black	7 (3.7)
Asian	0 (0.0)
Other	57 (30.2)
Hispanic or Latino ethnicity, n (%)	104 (55.0)
Weight in kg, median (range)	80 (46–120)
BMI, kg/m^2^, median (range)*	28.7 (16.5–45.3)
Duration of psoriasis, median years (range)†	15 (1–46)
Patients with prior psoriasis therapy, n (%)	158 (83.6)
Patients with prior systemic therapy, n (%)	153 (81.0)
PASI score, median (range)†	22 (7–61)
PASI score ≥ 20, n (%)†	111 (59.0)
NAPSI score > 0, n (%)	112 (59.3)
PSSI score > 0, n (%)	172 (91.0)
PPPASI score > 0, n (%)	19 (10.1)

* N = 180.

† N = 188.

BMI = body mass index; ITT = intent-to-treat; NAPSI = Nail and Psoriasis Severity Index; PASI = Psoriasis Area and Severity Index; PPPASI = Palmoplantar Pustulosis Psoriasis Area and Severity Index; PSSI = Psoriasis Scalp Severity Index.

### Treatment

The mean (SD) time on study (including follow-up) for the entire treatment group was 202 (52.9) days and the mean (SD) time on treatment was 139 (45.1) days. Almost two-thirds of patients (122/189 [64.6%]) received all 24 planned weekly injections; the mean (SD) number of injections received was 20 (6.4).

In the ITT population, 48.7% (92/189; 95% CI: 41.6–55.8%) of patients achieved or maintained a PGA score of “excellent” or “cleared” at Week 24. Using the nonresponder imputation, 46.0% (87/189; 95% CI: 38.9–53.1%) achieved this endpoint.

**Figure 1:**
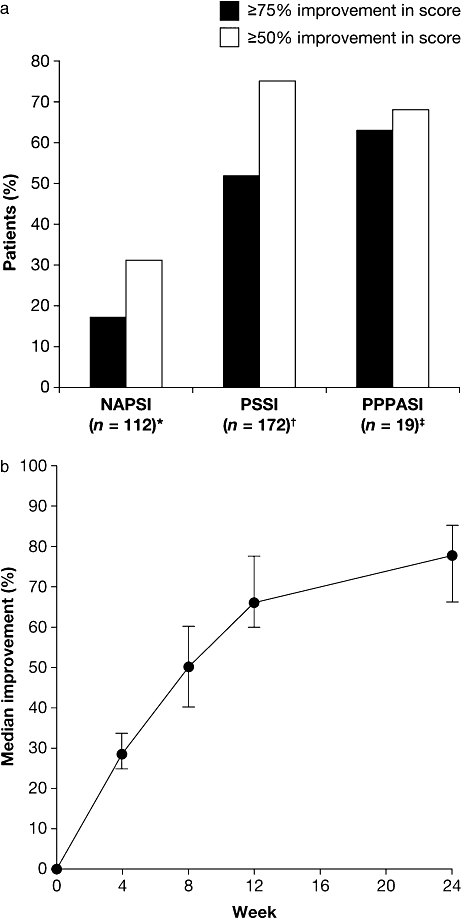
(a) Proportion of patients with at least 50% and 75% improvement in Nail Psoriasis Severity Index (NAPSI), Psoriasis Scalp Severity Index (PSSI), and Palmoplantar Pustulosis Psoriasis Area and Severity Index (PPPASI) scores at Week 24. Patients with: *NAPSI > 0 at baseline, ^†^PSSI > 0 at baseline, and ^‡^PPPASI > 0 at baseline. (b) Median (95% confidence interval) percentage improvement in Psoriasis Scalp Severity Index (PSSI) scores over time (N = 172) among patients with a PSSI score of >0 at baseline.

At baseline, 112 patients had nail psoriasis, with a median (range) score on the NAPSI of 14 (1–80) (Table 2). At Week 12, the median percentage improvement in the NAPSI was 0. At Week 24, the median percentage improvement from baseline in the NAPSI was 14.3% (95% CI: 0–29.4%; 32 missing values imputed). At this time point, 31% of patients with nail disease at baseline had achieved at least 50% improvement in the NAPSI score and 17% had at least a 75% improvement (Figure 1a).

**Table 2:** Median (interquartile range) Nail and Psoriasis Severity Index (NAPSI), Psoriasis Scalp Severity Index (PSSI), and Palmoplantar Pustulosis Psoriasis Area Severity Index (PPPASI) scores at baseline and at Week 24 among Latin American patients with scalp, palmoplantar and nail psoriasis who received subcutaneous efalizumab 1.0 mg/kg/wk in an open-label, noncomparative study

	Baseline		Week 24 visit	
Measure (sample size)	No imputation	LOCF	No imputation	LOCF
NAPSI (N = 112)	14.0 (6.0–27.0)	14.0 (6.0–27.0)	10.0 (2.0–17.0)	11.0 (4.0–20.0)
PSSI (N = 172)	16.0 (6.0–30.0)	16.0 (6.0–30.0)	3.0 (0.0–6.0)	3.0 (1.0–8.0)
PPPASI (N = 19)	2.0 (1.0–6.0)	2.0 (1.0–6.0)	0.0 (0.0–0.0)	0.0 (0.0–2.0)

LOCF = last observation carried forward.

Among patients with nail involvement at baseline, 31% had a NAPSI score of ≤7, 22% had a score of >7 to ≤14, 14% had a score of >14 to ≤21, 11% had a score of >21 to ≤28, and 21% had a score of >28. The proportions of patients in these categories showing at least a 75% improvement in NAPSI score at endpoint were 26%, 24%, 6%, 8% and 8%, respectively, indicating that rates of ≥75% improvement on the NAPSI were lower when the baseline score was higher. Corresponding proportions of patients in the categories defined by baseline score who showed at least a 50% improvement in NAPSI score at endpoint were 37%, 32%, 19%, 17% and 38%, respectively.

A total of 172 patients had scalp disease at baseline, with a median (range) PSSI score of 16 (1–54) (Table 2). At Week 24, the median percentage improvement from baseline in PSSI score was 77.6% (95% CI: 66.7–85.7%; 46 missing values imputed). At this time point, 71% of patients with scalp psoriasis at baseline had achieved at least 50% improvement in the PSSI score and 52% showed at least a 75% improvement (Figure 1a). These proportions were 58% and 43%, respectively, when the 46 patients with missing data were considered as nonresponders. An improvement in PSSI score was evident as early as Week 4 of treatment, and continued to increase throughout the treatment period (Figure 1b).

At baseline, the proportions of patients with PSSI scores of ≤7, >7 to ≤14, >14 to ≤21, >21 to ≤28, and >28 were 30%, 13%, 17%, 12% and 27%, respectively. The proportions of patients in these categories who showed at least a 75% improvement in PSSI score at Week 24 were 29%, 70%, 57%, 75% and 55%, respectively. The proportions of patients in the categories defined by baseline score who showed a 50% improvement in PSSI were 52%, 83%, 83%, 85% and 72%, respectively. These findings indicate that baseline PSSI scores >7 were associated with higher response rates than scores ≤7.

In 19 patients who had palmoplantar disease at baseline, the median (range) baseline PPPASI score was 2 (0–20) (Table 2). One patient had pustules at baseline, a corresponding PPPASI baseline score of 20.4, and a score of 0 at Week 24. At Week 24, the median percentage improvement from baseline in PPPASI was 100% (95% CI: 0–100%; 6 missing values imputed). At this time point, 68% of patients showed at least a 50% improvement and 63% showed at least a 75% improvement in PPPASI (Figure 1a). An improvement in PPPASI score was seen early in treatment, at Week 4 (51.0%; 95% CI: 0–83.3%), and continued to improve throughout the treatment period (91.7% [95% CI: 71.9–100%] at Week 8 and 97.5% [95% CI: 51.0–100.0%] at Week 12).

Among these 19 patients, the baseline PPPASI score was <2 for 11 patients, and between 2 and 8 for 5 patients; 3 patients had high scores (19.2, 19.6 and 20.4). Of those with low baseline scores (<2), 6 achieved a score of 0 on the PPPASI, 3 experienced worsening, and 2 withdrew from the study without an on-treatment PPPASI evaluation. Of the 5 patients with a baseline PPPASI score in the range 2–8, 4 had no psoriasis involvement after 24 weeks of treatment, whereas the remaining patient experienced worsening. The 3 patients with high baseline scores on the PPPASI experienced improvements of 51%, 98% and 100%.

The safety and tolerability of efalizumab are reported elsewhere [16].

No new safety concerns were identified in this population.

## Discussion

To our knowledge, this was the first large, prospective, international, multicentre study of any biological therapy in patients with psoriasis in Latin America. It was also the first study to assess the efficacy of efalizumab in the treatment of nail, scalp and palmoplantar psoriasis using the NAPSI, PSSI, and PPPASI, although these measures were not the primary endpoint of the parent trial.

Scalp psoriasis, which is currently difficult to treat in clinical practice, showed particularly promising results with efalizumab treatment during this trial, as measured by change from baseline PSSI score. Although the PSSI results reported were less impressive when using a nonresponder imputation than the more optimistic LOCF approach, we believe that the difference between these approaches was due more to the level of control of the patient's overall psoriasis than missing values for PSSI scores, which improved early and continued to improve throughout the study period. In addition, given that this was one of the largest clinical trials to date reporting the effect of any treatment on nail psoriasis, it could be concluded that the improvement achieved in nail psoriasis was clinically meaningful, as approximately one-third of patients achieved at least a 50% improvement from baseline NAPSI score.

The results for nail and palmoplantar psoriasis in the present study are in agreement with those reported from a comparison of efalizumab with placebo in patients with hand and foot plaque psoriasis [20]. In that study, 48% of patients achieved a PGA rating of “clear”, “almost clear” or “mild” disease after 12 weeks of treatment with efalizumab.

As topical therapies are generally the first-line treatment for nail, scalp and palmoplantar psoriasis, and systemic treatments are generally reserved for refractory disease [6,8,21,22], systemic treatments have usually been assessed only in small clinical trials. Conventional systemic therapies, such as retinoids, ciclosporin and methotrexate, have proved effective against psoriasis in these locations, but their use is limited by contraindications and potential toxicities [8,21,23]. Reports of the use of biological therapies in patients with scalp, nail, hand or foot psoriasis have mainly been restricted to small clinical trials or case studies. The exception is infliximab, which has been reported to show good efficacy against nail psoriasis in a large, randomized, placebo-controlled study [24,25], as well as in smaller studies [26,27]. However, no such efficacy has been reported for infliximab against palmoplantar or scalp psoriasis. Indeed, *de novo* manifestation of palmoplantar and scalp psoriasis has been reported during treatment with infliximab [28–30]. Clinical activity against nail psoriasis has been reported for alefacept in small, open-label trials and case series [31–33], and for etanercept in a case study [34]. In a small, open-label study, alefacept has been reported to have some efficacy against scalp psoriasis [35] and this agent has also shown promising activity against palmoplantar psoriasis in case studies [36,37] and small open-label trials [38,39]. In a small randomized clinical trial, some clinical activity against palmoplantar disease was reported with etanercept [40] and, in a retrospective study, etanercept was reported to reduce nail psoriasis during initial and repeated treatments [41]. Larger clinical trials are needed to confirm these findings.

The encouraging results from the current study are limited by the fact that this was an open-label trial with no comparator group, and that it involved patients with a wide range of disease severity affecting the nails, scalp and palmoplantar regions. Many of the patients also had relatively low baseline scores on the NAPSI, PSSI and PPPASI. For example, in other studies a NAPSI score of 15 has been considered to represent moderate-to-severe disease [32,42], whereas in the present study the median baseline score was 14. Furthermore, low baseline scores might have limited the treatment effects seen in individual patients and the median change seen in the group overall (or, in the case of the PPPASI, produced an unusually large treatment effect in patients with low baseline scores). However, despite these limitations, this study provides evidence of the potential of efalizumab as a treatment for nail, scalp and palmoplantar psoriasis. Large controlled studies would be needed to confirm these initial results. However, opportunistic infections have been reported in the post-marketing surveillance in patients with psoriasis receiving efalizumab. In particular, cases of JC virus infection resulting in progressive multifocal leucoencephalopathy have been reported in patients receiving efalizumab continuously for more than 3 years. After evaluating all available safety data, the European Medicines Agency concluded that the benefits of efalizumab treatment no longer outweighed the risks associated with the drug and recommended suspension of marketing authorisation on 19 February 2009. The drug has also been voluntarily withdrawn from the US market.
